# A Medical Aspect of the Education Proposal

**Published:** 1917-05-05

**Authors:** 


### A MEDICAL ASPECT OF THE EDUCATION PROPOSALS.

### By a Tuberculosis Worker.

THAT art is long and life short has always been one of the main difficulties in the path of human culture. Especially has it been an obstacle to educationists, as that term is usually taken, because in place of the whole period of life only a portion is available to them. As knowledge increases they have had to claim more and more years in which to teach it. All curricula have had to be lengthened, our own medical one included. A few excisions can be made—it is sad, for instance, to think of the time mankind has wasted upon theology—but for the place of each omission many fresh subjects compete. It is not surprising, therefore, to see, now that the nation has had its eyes opened to its educational leeway, another claim on the time of the nation's children. As was foreshadowed in THE HOSPITAL of April 7, Mr. Fisher has proposed the establishment of " nursery schools," for infants from two to six years, ages which cover the present pre-educational period, which begins ordinarily, that is compulsorily, at five years old. In all probability the true source of this proposal may be traced, as stated previously in this Journal, to the Workers' Educational Association, a body federating many working-class organisations and voicing their educational demands.

DISCLAIMER NO. 1.

Now let no one think that the present writer yields to anyone in solicitude for the children of the proletariat. Old Jonas Hanway's title, " Letters on the Importance of the rising Generation of the labouring part of our Fellow-Subjects," raises a keen thrill of approval, and feelings of regret that comparatively so little has been done, in the century and a half that has elapsed since he wrote, in the direction he so wisely indicated. The strength of Germany has arisen not a little from its national insurance measures and its educational policy, and the divorce it accomplished between those too tightly wedded phenomena, industrialism and squalor. It is because of the utmost sympathy with the object of these nursery schools, which may be taken to be the bodily and mental amelioration of young working-class children, that one doubts the wisdom of establishing them except under conditions that have nowhere transpired, and probably have never been under consideration. Quite rightly, in the case of infants, development and care have been stressed rather than instruction; that is, bodily rather than mental culture. This, since the days of the kindergarten, is an accepted principle. But the fact is that all schooling, except mild open-air schooling, is a strain upon the health, certainly of a working-class, probably of any, child; above all, upon the health of a very young child. In school medical inspection, and in dispensary tuberculosis work, again and again and again does one see children falling ill soon after beginning school attendance; or falling ill, to recover when kept at home, to fall ill again soon after going back—this process going on perhaps for years, for all the world like a recurring decimal of two figures; or keeping well indefinitely without any medical treatment, so long as at home, perhaps for as much as two years, only to relapse, and stay unwell spite of all drugs can do, as long as school attendance goes on; and when it ceases, the little one is restored to health and to duncehood. Working-class parents are often unjustly blamed for detaining their offspring from school in order to make use of them at home. The fact of the matter often is that mothers come to see for themselves that their children's health is suffering from that cause alone.

DISCLAIMER NO. 2.

Let no one again think that this is the fault of our Council and Borough schools. Compared with the old Board schools of thirty years ago, they are admirably planned structures, light, sunny, full of flowers and cheerful pictures and simple artistic objects. Nor is it the fault of the teachers, of which statement the best possible proof is that the children dislike staying away. That is a change educationists have not made enough capital out of, the disappearance of the Shakespearean " creeping like snail unwillingly." The children like their schooling very much. If these infant schools were established *tout court*, they would have excellent attendances. For, in the case of such young scholars, every mother and father would like well enough, too, to have the crowded home cleared, and the overworked housekeeper relieved of responsibility, for so many hours every day. How well it sounds *a priori*—infants properly looked after, taken out of mean surroundings, saved from the dangers of the street and the slum! And how very badly it would work out in practice! For the conclusion to which clinical observation, the only decisive test where bodily amelioration is the main avowed object, points is that playing in the streets in childhood is the very saving, the very making, of the national physique.

THE BALANCE OF ADVANTAGE.

Hop-scotch in the gutter makes and saves the proletarian's constitution in the many severe trials it is subject to, at some cost, however. There is the risk, nowadays, from automobiles; there is the summer dust; there are in slum neighbourhoods unedifying street scenes. But the child does get complete freedom in the open air, like a puppy at " walk " (which means that he walks where he likes), like a lamb in the fields. All stock-breeders—and in sport and stock-breeding England leads the world—give their young animals life-conditions as feral as possible. Our national schools, however well managed, can only give an inevitable restraint and an indoor atmosphere. In a northern climate like that of England an indoor atmosphere is very different from an outdoor one. It is dry, charged with dust, warm and stagnant, two qualities, these last, which, as Professor Leonard Hill's work has shown, are particularly deleterious to health. In fine, if these new infant schools are to be on the model of those already started in some places, the prediction is here made that they will on balance injure the national health. If, on the contrary, they are constituted as open-air crèches, and no attempt be made to inject knowledge into the scholars, good will result, probably returning the value of the money expended: for a clinician to hazard conjectures as to whether the limited funds had not better be saved for continuation schooling at the present leaving age would be unwarrantable. To return, these nursery schools, if started, should be on the model of open air or "forest" schools, institutions of which many too few exist. Quite recently a large industrial district like Tyneside did not possess either. The forest school is a very valuable institution, on account of the protection from the weather which is secured, but large pine woods near a thickly-populated poor neighbourhood, such as those made use of at Bostal Wood, near Plumstead, do not often occur. It may come to planting quick growing conifers, such as Scotch fir or *pinus insignis*, in the requisite localities—a better and more creditable investment than game covers, which before the war were being planted every day. And above all, there should be no routine or restraint or flag-waving or drilling or "exercises"; prizes might be awarded for mud-pies, but for nothing else.

